# Transbrachial Access for Transcatheter Closure of Paravalvular Leak Following Prosthetic Valve Replacement

**DOI:** 10.3389/fcvm.2021.589947

**Published:** 2021-02-26

**Authors:** Hui Zhang, Jing-Yan Wang, Jian-Hua Lv, Hai-Bo Hu, Rui-Gang Xie, Qi Jin, Kun-Jing Pang, Liang Xu, Zhong-Ying Xu, Ge-Jun Zhang, Xiang-Bin Pan

**Affiliations:** ^1^Department of Radiology, Zhengzhou University People's Hospital, Central China Fuwai Hospital, Heart Center of Henan Provincial People's Hospital, Zhengzhou, China; ^2^Department of Cardiology, Yuncheng Central Hospital, Yuncheng, China; ^3^Center of Structural Heart Disease, National Center for Cardiovascular Diseases, Chinese Academic of Medical Science and Peking Union Medical College, Fuwai Hospital, Beijing, China; ^4^Center for Pulmonary Vascular Diseases, National Center for Cardiovascular Diseases, Chinese Academy of Medical Sciences and Peking Union Medical College, Fuwai Hospital, Beijing, China; ^5^Department of Echocardiography, National Center for Cardiovascular Diseases, Chinese Academic of Medical Science and Peking Union Medical College, Fuwai Hospital, Beijing, China

**Keywords:** prosthetic valve replacement, paravalvular leak, brachial artery approach, transbrachial access, transcatheter closure

## Abstract

**Background:** Transcatheter closure of paravalvular leak (PVL) has evolved into an alternative to surgery in high-risk patients. In this study, we introduce a new access for transcatheter closure of PVL and seek to evaluate the feasibility and safety of this access.

**Methods:** We retrospectively analyzed patients undergoing transbrachial access for transcatheter mitral or aortic PVL closure (August 2017–November 2019) at our hospital. All patients underwent puncture of the brachial artery under local anesthesia.

**Results:** The study population included 11 patients, with an average age of 55.91 ± 14.82 years. Ten out of 11 patients were successfully implanted with devices *via* the brachial artery approach, and one patient was converted to the transseptal approach. The technical success rate of transbrachial access was 90.9%. Mean NYHA functional class improved from 3.1 ± 0.5 before the procedure to 1.9 ± 0.5 after PVL closure. Severe paravalvular regurgitation (PVR) in five patients and moderate PVR in six patients prior to the procedure were significantly reduced to mild in four patients and none in seven patients after the procedure. Complications included one case of pseudoaneurysm and one case of moderate hemolysis aggravation after closure. One patient had an unknown cause of sudden death within 24 h after the procedure. The half-year mortality rate during follow-up was 9.1% (1/11).

**Conclusions:** Transbrachial access for transcatheter closure of PVL may be a feasible and safe treatment and should include well-selected patients. It has several potential advantages of simplifying the procedure process and reducing postprocedural bed rest time.

## Introduction

Paravalvular leak (PVL) is a common and challenging complication following prosthetic valve replacement. Approximately 2–5% of PVLs are associated with congestive heart failure (HF), hemolytic anemia, and infective endocarditis and need further surgical or interventional treatment (1, 2).

Since transcatheter closure of PVL was first reported in 1992 (3), it has been developed into a feasible alternative to repeat surgery in recent years (4, 5). Traditionally, transcatheter closure of PVL usually is performed *via* several routes including the transfemoral, transseptal, and transapical approaches. However, the three traditional routes all need to stop warfarin before the procedure and need bed rest and activity restriction after the procedure. Another potential problem is that the working distance of the standard sheath is too short to reach the level of the mitral valve in taller patients *via* the transfemoral or transseptal approach.

Transbrachial access, by contrast, can be an alternative route to transcatheter closure of the PVL in this setting. To minimize the invasiveness of the procedure and reduce bed rest time, we introduced transbrachial access for transcatheter closure of PVL. Thus, the purpose of our study was to evaluate the feasibility and safety of transbrachial access for transcatheter closure of paravalvular leak following prosthetic valve replacement.

## Methods

### Patient Population

Between August 2017 and November 2019, 11 procedures with transbrachial access were performed in 11 patients undergoing transcatheter closure of mitral or aortic PVL. Seven patients (63.6%) were males, and the mean age was 55.91 ± 14.82 years. The indications for PVL closure were: (I) moderate to severe paravalvular regurgitation, (II) severe symptoms of dyspnea or clinically significant. Hemolytic anemia, and (III) congestive heart failure (NYHA functional class III to IV); Patients were excluded for the following: (I) active endocarditis, (II) prosthetic valve with thrombi or vegetation, (III) unstable or rocking prosthesis, (IV) patients with significant dehiscence involving more than one-fourth to one-third of the valve ring, and (V) prosthetic dysfunction. The demographics and comorbidities were collected through the electronic medical record (EMR). The study was approved by the Ethics Committee of Fuwai Cardiovascular Hospital. Patients were advised of the procedural risks and options as well as the off-label use of the closure devices, and all patients signed informed consent.

### Pre-operative Diagnosis and Imaging Evaluation

The patients received transthoracic echocardiography (TTE), transoesophageal echocardiography (TEE), and computed tomography (CT, if necessary) before the procedure to define the location, size, shape, and trajectory of PVL. The grading of paravalvular regurgitation was performed using a 3-class grading scheme (mild, moderate, and severe) that was assessed with color flow Doppler. For mitral and aortic PVL, the circumferential extent of PVL was classified as mild (<10%), moderate (10–30%), and severe (>30%) (6, 7).

In view of the medical costs and patients being unable to tolerate TEE for a long time, TTE (a less invasive procedure) was selected for intraprocedural monitoring and post-procedural follow-up.

### Procedure

All patients took medication to improve cardiac function before the procedure. Warfarin was continued without heparin bridging during the perioperative period, with a view to a transbrachial procedure. The procedures were performed under local anesthesia in a catheterization room with X-ray fluoroscopic and TTE guidance. Retrograde access *via* the brachial artery approach was adopted. Fluoroscopy confirmed the presence of paravalvular regurgitation, and TTE determined the location of the PVL. Heparin (100 U/kg, Sinopharm Chemical Reagent Co. Ltd, China) was introduced *via* the sheath.

#### Puncture of the Brachial Artery

The patient's right upper limb was placed on the support plate of the operation table with the palm upward and slightly abducted. The arterial puncture point was performed ~1.0 cm horizontally below the elbow joint on the right upper limb. A 5F, 6F, or 7F vascular sheath with a hemostatic valve was placed after the puncture was completed.

#### Mitral PVL Procedure

The diagnostic catheter was introduced from the brachial artery to the subclavian artery, aortic arch, ascending aorta, and then placed in the left ventricle. A hydrophilic guidewire with straight tip (Terumo, 0.035″ 260 cm) was passed through the diagnostic catheter (Judkins right coronary catheter, or a cut pigtail catheter), used to cross the PVL into the left atrium and then advanced into the pulmonary vein or repeatedly circled and fixed into the left atrium The diagnostic catheter was then removed, followed by a 6F or 7F delivery sheath over the guidewire into the left atrium.

After introducing the delivery sheath into the left atrium, the dilator catheter was withdrawn from the delivery sheath, leaving the delivery sheath in the left atrium. A second hydrophilic guidewire with straight tip (Terumo, 0.035″ 260 cm) was passed through the delivery sheath and then advanced into the pulmonary vein or repeatedly circled and fixed into the left atrium. The delivery sheath was then withdrawn from the body, leaving only two guidewires across the leaks in the left atrium.

The dilator catheter was then assembled into the delivery sheath after heparin flushing again *in vitro*. Next, the entire delivery sheath assembly was advanced into the left atrium over one of the two guidewires. The dilator catheter and the guidewire that was in the delivery sheath were then removed together. Finally, the device (previously selected) was advanced through the delivery sheath and then deployed. Now, the second hydrophilic guidewire was still left in the left atrium outside of the delivery sheath [Modified double wire technique].

**Figure 1:**
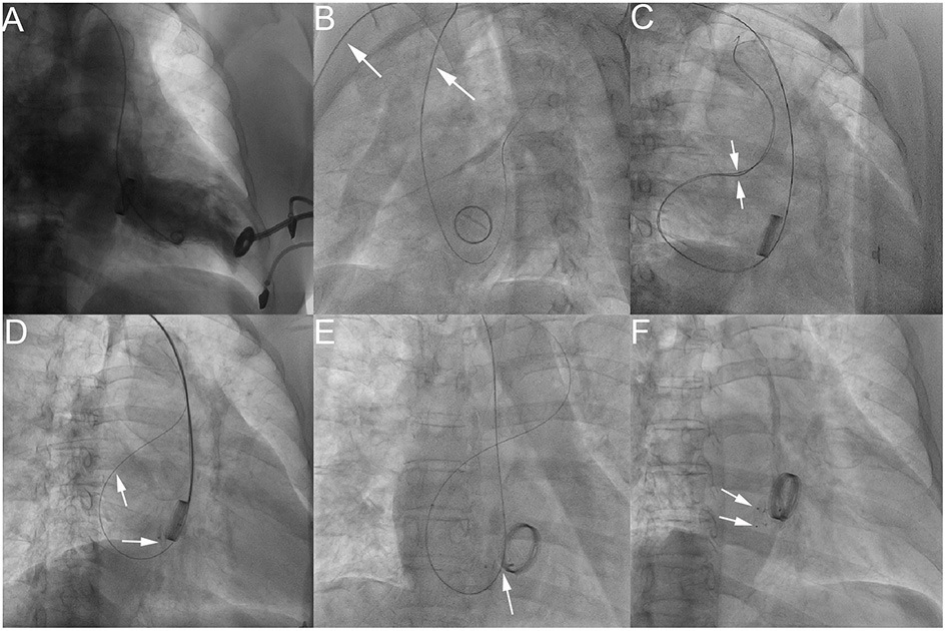
The process of transcatheter closure of mitral PVL. LV angiography in AP projection shows severe mitral paravalvular regurgitation **(A)**; LAO 50° projection shows that a hydrophilic guidewire was introduced retrogradely from the subclavian artery (white arrow), aortic arch, ascending aorta (white arrow), into the left ventricle and then crossed the mitral paravalvular leak into the left atrium **(B)**; Two hydrophilic guidewires were passed through the delivery sheath and then advanced into the left superior pulmonary vein **(C)**; The first device was released through the delivery sheath over the first guidewire with the second hydrophilic guidewire leaving outside of the delivery sheath in the left atrium **(D)**. The AP projection shows that the second device was released after introducing the delivery sheath over the retained guidewire (the second hydrophilic guidewire) **(E)**. The RAO 30° projection shows that two devices were deployed **(F)**. −PDA occluder (Starway Medical Technology, Inc., Beijing, China).

If the regurgitant jet of the PVL under the TTE was more than 3 mm after releasing the first device, another device could be loaded into the delivery sheath which could be advanced into the left atrium over the second guidewire. According to this method, multiple devices could be implanted sequentially until it was confirmed that the occlusion effect was satisfactory (Figure 1).

#### Aortic PVL Procedure

**Figure 2:**
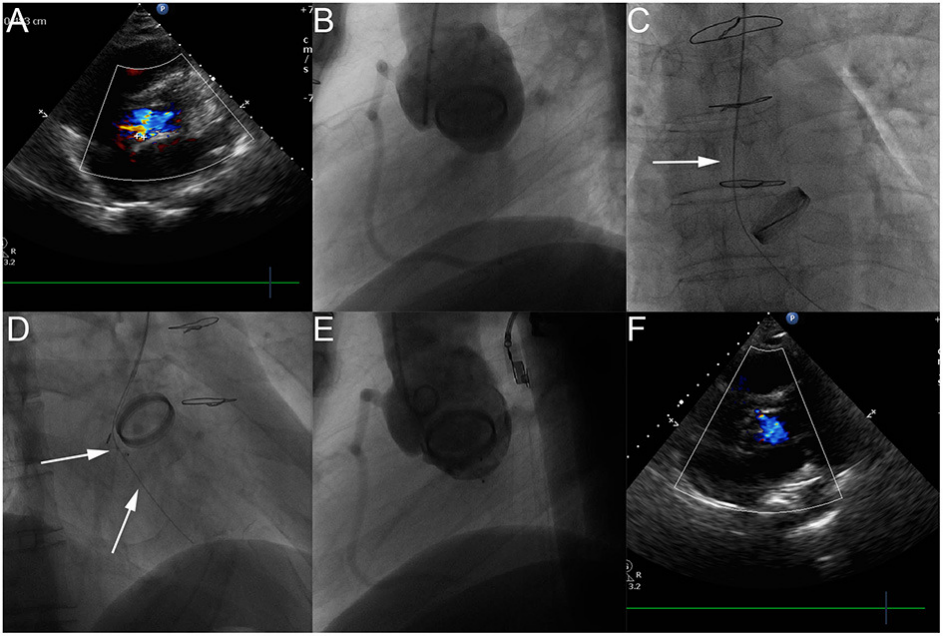
The process of transcatheter closure of aortic PVL. Transthoracic echocardiography (TTE) shows moderate aortic paravalvular regurgitation **(A)**. The LAO 50° projection shows that moderate aortic paravalvular regurgitation **(B)**. The AP projection shows that a hydrophilic guidewire was introduced retrogradely from ascending aorta (white arrow), crossing the aortic PVL, and finally going into the left ventricle **(C)**. The RAO 30° shows that the first device was released through the delivery sheath over the first guidewire with the second hydrophilic guidewire leaving outside of the delivery sheath in the left ventricle **(D)**. The LAO 50° projection shows that aortic paravalvular regurgitation disappeared **(E)**. TTE shows that aortic paravalvular regurgitation disappeared after the procedure **(F)**.

The diagnostic catheter was introduced from the brachial artery to the subclavian artery, aortic arch, and ascending aorta. A hydrophilic guidewire with straight tip (Terumo, 0.035″ 260 cm) was passed through the diagnostic catheter [Judkins right coronary catheter, or 5F MPA2 [Cordis]], used to cross the PVL from the aortic root into the left ventricle and then repeatedly circled and fixed into the left ventricle. The diagnostic catheter was then removed, followed by a 5F, 6F, or 7F delivery sheath over the guidewire into the left ventricle. Next, one or more occluder devices were deployed using the “*Modified double wire technique*” mentioned above (Figure 2).

Because of the minimally invasive puncture of the upper limb only, warfarin was continued before, during, and after the procedure with no need for heparin bridging. The patients were discharged about 48–72 h after the procedure.

### Follow-Up

In all patients, TTE, ECG, chest radiograph, and laboratory examination (if necessary) were evaluated at discharge and 1, 3, and 6 months after the procedure, as well as every 1 or 2 years thereafter. Procedural success was defined as specified in a recent expert statement paper (8). Clinical success was defined as an improvement of at least one NYHA functional class with no rehospitalizations or reinterventions for the index reason (8). Follow-up was achieved using a telephone directly or at an outpatient clinic of our hospital.

## Results

A total of 11 patients were enrolled. The median time from the last surgical valve replacement to transcatheter closure attempt was 3.0 (0.8–14) years. Eight out of eleven (72.7%) patients' had PVLs occurs within the first year of valve implantation, which is in accordance with previously published reports (9). There were eight cases with aortic PVL, and three cases with mitral PVL. The patients' data are detailed in Table 1.

**Table 1:** Patients' data.

**Patient**	**Age**	**Clinical manifestations**	**Target valve**	**Pre-op. NYHA**	**Pre-op. PVL grade**	**Delivery Sheath size**	**Post-op. PVL grade**	**Operation time (min)**	**Fluoroscopy time (min)**
1	46	HF	Aortic	III	Moderate	5F	None	60	15
2	46	HF	Aortic	III	Moderate	5F	None	70	18
3	69	HF+HA	Mitral	III	Severe	6F	Mild	115	20
4	62	HF+HA	Mitral	III	Severe	7F	Mild	130	25
5	60	HF+HA	Aortic	IV	Moderate	6F	None	150	26
6	31	HF	Aortic	III	Moderate	5F	Mild	60	12
7	73	HF+HA	Mitral	IV	Severe	7F	Mild	180	30
8	45	HF	Aortic	II	Moderate	5F	None	125	32
9	70	HF	Aortic	III	Severe	5F	None	130	25
10	73	HF+HA	Aortic	III	Severe	5F	None	90	15
11	40	HF	Aortic	III	Moderate	5F	None	200	50

*NYHA, New York Heart Association; HF, Heart failure; HA, Hemolytic anemia*.

10 out of 11 patients had transcatheter closure of PVL performed *via* the brachial artery successfully. Because of finding a small atrial septal defect (ASD) under TTE with color flow Doppler, patient 7 was converted to “transseptal access” for interventional closure. The technique success of transcatheter closure of PVL *via* the brachial artery approach was 90.9% (10/11). The delivery sheath sizes ranged from 5F to 7F. The average procedural time and fluoroscopy time was 119.1 ± 46.63 min and 24.36 ± 10.67 min, respectively.

Patient 11 with aortic PVL had an unknown cause of sudden death within 24 h after the procedure. A case of moderate hemolysis aggravation occurred in patient 7, and he was successfully discharged after dialysis treatment. Patient 3 had a brachial pseudoaneurysm on the second day and was discharged smoothly after being treated with a thrombin injection under ultrasound guidance. The clinical success rate of this group of patients was 81.8% (9/11). Median NYHA functional class improved from 3.1 ± 0.5 before the procedure to 1.9 ± 0.5 after PVL closure. Severe PVR in five patients and moderate PVR in six patients prior to the procedure were significantly reduced to mild in four patients and none in seven patients after the procedure. The average hospital stay was 7.2 ± 4.5 days. The clinical outcomes are detailed in Table 2.

**Table 2:** Clinical outcome (in-hospital, 30-day and half-year follow-up).

Patients treated: *n* = 11	
In-hospital follow-up:	
Clinical success	81.8% (9/11)
ICU time (days, median, IQR)	0 (0, 1)
Hospital stay (days, mean)	7.2 ± 4.5
NYHA functional class post-procedure	
NYHA class IV	0% (0/11)
NYHA class III	9.1% (1/11)
NYHA class II	72.7% (8/11)
NYHA class I	18.2% (2/11)
Complications	
Hemothorax	0% (0/11)
Arterial pseudo-aneurysm	9.1% (1/11)
Major bleeding requiring blood transfusion	0% (0/11)
Device displacement	9.1% (1/11)
Cardiac perforation/cardiac tamponade	0% (0/11)
Moderate hemolysis aggravation	9.1% (1/11)
Prosthetic leaflet impingement by plug	0% (0/11)
Conversion to surgery	0% (0/11)
Stroke	0% (0/11)
In-hospital mortality rate	9.1% (1/11)
The in-hospital mortality rate for cardiovascular cause	9.1% (1/11)
30-day mortality rate (all-cause)	9.1% (1/11)
Half-year mortality rate (all-cause)	9.1% (1/11)

There were no new deaths within 30 days of discharge.

## Discussion

Currently, transfemoral or transseptal routes are the mainstay of access for percutaneous closure of PVL. However, the feasibility and safety of transbrachial access for transcatheter closure of paravalvular leak remain uncertain. In daily clinical practice, transfemoral and transseptal routes are not always possible, particularly in patients who have severe occlusive peripheral vascular disease (10, 11). As for transfemoral access, the working distance of the standard sheath is sometimes too short to reach the level of the mitral valve in taller patients. Percutaneous puncture of the femoral artery has a high access site complication rate in fully anticoagulated patients (12). For transseptal access, the atrial septum after surgical valve replacement is often surgically reinforced, so it is difficult to puncture. Hence, we actively explored transbrachial access, a novel approach for transcatheter closure of PVL.

In this small group of cases, we find that the technical success of transbrachial access for transcatheter closure of PVL is 90.9%. We can close most PVLs (width <10 mm, and length of <15 mm) *via* the brachial artery approach. Several previous studies have reported that the clinical success rate of transcatheter closure of PVL ranged from 69.5 to 93% (13–15). In our study, nine patients achieved clinical improvement. The clinical success rate of our small group of cases was 81.8%, which was close to the previous study. Hence, transbrachial access for transcatheter closure of paravalvular leak is a feasible and efficacy treatment.

Transbrachial access offers potential advantages over transfemoral or transseptal access. (I) The transbrachial access can improve patients' comfort after closure, since ambulation is permitted immediately after the procedure. This reduces the additional risk of pulmonary embolism and shortens the hospital stay. Except for the one patient who had a moderate hemolysis aggravation after the procedure, the average hospital stay of the other patients after the intervention was only 4.4 days, which is very advantageous compared with previous studies. (II) Warfarin was continued before and during the procedure, which reduces the risk of thrombus blocking the prosthetic valve. (III) The distance from the brachial artery to the level of the mitral annulus was much shorter than that of the femoral artery, avoiding the situation where the delivery sheath is not long enough for PVL closure. (IV) Due to the relatively small size of the delivery sheath (5F−7F) and the hydrophilic guidewire used as an exchange wire, the risk of PVL tearing and vascular injury, which was introduced by previous Amplatz Extra Stiff wire and large size of the delivery sheath, can be minimized. (V) Transbrachial access can also be used as an alternative approach for transfemoral access with poor conditions. (VI) We punctured the brachial artery (not the femoral artery), reducing the risk of fatal retroperitoneal hematoma created by high-level femoral artery puncture (16, 17). By contrast, the brachial artery is more superficial, and the hematoma is easier to find and deal with immediately. For example, one case with brachial artery pseudoaneurysm in this group was treated in a timely manner.

Transvenous access with transseptal puncture is commonly used for anterograde access to mitral PVL. Nevertheless, in this study, transbrachial access was used for retrograde closure of mitral PVL. Transbrachial access has the risk of interfering with aortic valve, but no interference with aortic valve was found in this study. This requires strict standardized procedure and closely intraoperative monitoring with ultrasound to avoid this risk. Transfemoral arterial access is commonly used for some mitral leaks in a medial position (5). Besides, Gafoor (18) recommend approaching mitral leaks first using a retrograde transfemoral access. Transfemoral access avoids puncturing the atrial septum. If the retrograde transfemoral approach is unsuccessful in crossing the leak, an antegrade transseptal approach is used. In this sense, transbrachial access for transcatheter mitral PVL closure has potential advantages over a retrograde transfemoral access.

In our experience, the integration of the modified double wire technique has been very useful for the deployment of multiple devices *via* the brachial artery approach, which can simplify the process and achieve a more satisfactory occluder effect. Large PVLs usually need multiple occluder devices. When the deployment of multiple devices is planned, the “double wire–simultaneous deployment” or “anchor, single wire technique–sequential deployment” technique is selected (19, 20). The “double wire-simultaneous deployment” technique has the advantages that two devices are released simultaneously with good coordination, which can make devices more stable, but two delivery sheaths being simultaneously introduced into the access vessels can damage these vessels. For the “anchor, single wire technique-sequential deployment” technique, there is only one delivery sheath introduced into the access vessels. However, the second attempt to cross the PVL may lead to the displacement of the first occluder. Meanwhile, since the delivery sheath has been removed, the first occluder can't be recycled immediately. Hence, in our study, the new modified double wire technique was applied. Because the brachial artery has a much smaller average diameter compared to the femoral artery, the two techniques above are not appropriate for the transcatheter closure of PVLs *via* the brachial artery approach. The “modified double wire technique” approach is a very useful technique with potential advantages and should be considered in a similar clinical situation.

In our study, complications were observed in three patients. One case had an unknown cause of sudden death within 24 h after the procedure, and an autopsy was not performed. We consider the possible reasons for this as follows: acute obstruction of the left coronary ostium due to occluder displacement, or acute intracoronary thrombosis. A second case was complicated by a brachial artery pseudoaneurysm on the second day, which was due to insufficient compression of the right upper limb puncture site. Therefore, we believe that the interventionalist should pay attention to the intraoperative and postoperative management of the access site to reduce related vascular complications and the risk of ischemia to the arm. A third case had moderate hemolysis aggravation after the procedure. We propose that the mVSD (with blocking-flow membrane) occluder is related to the outcome of hemolysis aggravation. Recent work has established that mVSD is an independent risk factor for hemolysis aggravation (13). The blocking-flow membrane of the mVSD is usually made of nitinol mesh, which may increase flow turbulence and shear stress. This can be considered to explain hemolysis aggravation. Therefore, we suggest reducing the use of occluder devices with blocking-flow membranes in clinical practice.

### Limitations

Our present study had several limitations. First, this is a preliminary clinical study with a relatively small number of patients. Multicenters, large sample sizes of patients, and mid- or long-term follow-up studies are needed. Second, since the brachial artery route can only pass through a delivery sheath with a size of 5F−7F, a large PVL (a width >10 mm) is not suitable for this access. Therefore, the methods may not be applicable to all patients. Third, because of the diversity and complexity of the leaks, as well as the need for a surgical view, it would be better if the 3D TEE modality for echocardiography guidance was used.

### Conclusion

Transbrachial access for transcatheter PVL closure may be feasible for well-selected patients presenting with a suitable size of PVL and has several potential advantages. Technique success for mitral PVL and aortic PVL closure *via* the brachial approach was high and was associated with significant improvement in NYHA class and transfusion dependency. However, a larger sample size with mid- and long-term follow-up is mandatory to assess the clinical outcome of patients treated with this access.

## Data Availability Statement

The raw data supporting the conclusions of this article will be made available by the authors, without undue reservation.

## Ethics Statement

The studies involving human participants were reviewed and approved by the Ethics Committee of Fuwai Cardiovascular hospital. The patients/participants provided their written informed consent to participate in this study. Written informed consent was obtained from the individual(s) for the publication of any potentially identifiable images or data included in this article.

## Author Contributions

H-BH, HZ, and J-HL: conception and design. X-BP, G-JZ, and Z-YX: administrative support. J-YW, K-JP, and H-BH: provision of study materials or patients. LX and R-GX: collection and assembly of data. H-BH, HZ, and QJ: data analysis and interpretation. All authors:manuscript writing and final approval of manuscript.

## Conflict of Interest

The authors declare that the research was conducted in the absence of any commercial or financial relationships that could be construed as a potential conflict of interest.

## Abbreviations

PVL: paravalvular leak
CABG: coronary artery bypass grafting
CHF: chronic heart failure
HF: heart failure
TEE: transesophageal echocardiography
CT: computed tomography
TTE: transthoracic ultrasound
NYHA: New York Heart Association
LVEF: Left ventricular ejection fraction.
